# Supplementary material for: Effects of Perineal Warm Compresses during the Second Stage of Labor on Reducing Perineal Trauma and Relieving Postpartum Perineal Pain in Primiparous Women: A Systematic Review and Meta-Analyses
Source: Healthcare (Basel). 2024 Mar 22;12(7):702. doi: 10.3390/healthcare12070702 (PMC11011582; doi:10.3390/healthcare12070702)
Supplement: Supplementary file 1 [file healthcare-12-00702-s001.zip › Supplementary File S1. Search strategy.pdf]

Supplementary File S1. Search strategy

Search date: from inception to March 15, 2023

Database/Register	Search strategies		Results
PubMed	#1	"Perineum"[MeSH Terms] OR "Vulva"[MeSH Terms] OR "Vagina"[MeSH Terms]	54,561
	#2	"perine*"[Title/Abstract] OR "vulva*"[Title/Abstract] OR "vagin*"[Title/Abstract] OR "genital tract*"[Title/Abstract] OR "reproductive tract*"[Title/Abstract] OR "birth canal"[Title/Abstract] OR "obstetric canal"[Title/Abstract] OR "parturient canal"[Title/Abstract]	202,412
	#3	#1 OR #2	217,212
	#4	"warm compress*"[Title/Abstract] OR "warm application"[Title/Abstract] OR "warm packs"[Title/Abstract] OR "warm"[Title/Abstract] OR "heat therapy"[Title/Abstract] OR "heat application"[Title/Abstract] OR "heat*"[Title/Abstract] OR "thermotherapy"[Title/Abstract] OR "physiotherapy"[Title/Abstract] OR "physical therapy" [Title/Abstract]	421,429
	#5	"labor, obstetric"[MeSH Terms] OR "delivery, obstetric"[MeSH Terms] OR "Parturition"[MeSH Terms]	137,491
	#6	"deliver*"[Title/Abstract] OR "birth"[Title/Abstract] OR "childbirth"[Title/Abstract] OR "labor"[Title/Abstract] OR "labour"[Title/Abstract] OR "parturition"[Title/Abstract] OR "obstetric*"[Title/Abstract] OR "intrapartum"[Title/Abstract]	1,282,638
	#7	#5 OR #6	1,321,730
	#8	#3 AND #4 AND #7	413
Embase	#1	perineum:ti,ab,kw OR vagina:ti,ab,kw OR perine*:ti,ab,kw OR vulva:ti,ab,kw OR vagin*:ti,ab,kw OR 'genital system':ti,ab,kw OR 'genital tract':ti,ab,kw OR 'reproductive tract':ti,ab,kw OR 'birth canal':ti,ab,kw OR 'obstetric canal':ti,ab,kw OR 'parturient canal':ti,ab,kw	276,634
	#2	'warm compress':ti,ab,kw OR 'warm application':ti,ab,kw OR 'warm packs':ti,ab,kw OR heat*:ti,ab,kw OR warm*:ti,ab,kw OR thermotherapy:ti,ab,kw OR 'heat application':ti,ab,kw OR physiotherapy:ti,ab,kw	505,555
	#3	'obstetric delivery':ti,ab,kw OR birth:ti,ab,kw OR childbirth:ti,ab,kw OR labor:ti,ab,kw OR 'intrapartum care':ti,ab,kw OR parturition:ti,ab,kw OR obstetric*:ti,ab,kw OR intrapartum:ti,ab,kw	709,605
	#4	#1 AND #2 AND #3	506
CINAHL Plus	#1	SU ((perineum OR vulva OR vagina OR perine* OR vulva* OR vagin* OR "genital tract*" OR "reproductive tract*" OR "birth canal" OR “obstetric canal” OR “parturient canal”))	21,428
	#2	SU ((“warm compress*” OR “warm application” OR “warm packs” OR warm* OR “heat therapy” OR “heat application” OR heat* OR thermotherapy OR physiotherapy OR “physical therapy”))	96,928
	#3	SU ((deliver* OR birth OR childbirth OR labor OR labour OR parturition OR obstetric* OR intrapartum))	239,263
	#4	#1 AND #2 AND #3	67
Web of Science	#1	TS=((perine* OR vulva* OR vagin* OR "genital tract*" OR "reproductive tract*" OR "birth canal" OR “obstetric canal” OR “parturient canal”))	186,016
	#2	TS=((“warm compress*” OR “warm application” OR “warm packs” OR warm* OR “heat therapy” OR “heat application” OR heat* OR thermotherapy OR physiotherapy OR “physical therapy”))	1,763,268
	#3	TS=((deliver* OR birth OR childbirth OR labor OR labour OR parturition OR obstetric* OR intrapartum))	1,820,560
	#4	#1 AND #2 AND #3	660
China National Knowledge Infrastructure (CNKI)	#1	SU = "会阴" + "外阴" + "阴道" + "产道" + "生殖道"	203,375
	#2	SU = "热敷" + "热疗" + "温敷" + "湿敷" + "物理疗法" + "物理治疗"	32,654
	#3	SU = "分娩" + "生产" + "产程" + "产时" + "产中"	3,765,449
	#4	#1 AND #2 AND #3	125
WanFang Data Knowledge Service Platform (WanFang Data)	#1	主题:("会阴") OR 主题:("外阴") OR 主题:("阴道") OR 主题:("产道") OR 主题:("生殖道")	255,032
	#2	主题:("热敷") OR 主题:("热疗") OR 主题:("温敷") OR 主题:("湿敷") OR 主题:("物理疗法") OR 主题:("物理治疗")	40,475
	#3	主题:("分娩") OR 主题:("生产") OR 主题:("产程") OR 主题:("产时") OR 主题:("产中")	4,169,440
	#4	题名:(剖宫)	78,398
	#5	(#1 AND #2 AND #3) NOT #4	389
Chinese Biomedical Literature Service System (SinoMed)	#1	"会阴"[不加权:扩展] OR "外阴"[不加权:扩展] OR "阴道"[不加权:扩展] OR "会阴"[常用字段:智能] OR "外阴"[常用字段:智能] OR "阴道"[常用字段:智能] OR "产道"[常用字段:智能] OR "生殖道"[常用字段:智能]	188,007
	#2	"热敷"[常用字段:智能] OR "热疗"[常用字段:智能] OR "温敷"[常用字段:智能] OR "湿敷"[常用字段:智能] OR "物理疗法"[常用字段:智能] OR "物理治疗"[常用字段:智能]	42,417
	#3	"分娩"[不加权:扩展] OR "分娩"[常用字段:智能] OR "生产"[常用字段:智能] OR "产程"[常用字段:智能] OR "产时"[常用字段:智能] OR "产中"[常用字段:智能]	530,318
	#4	#1 AND #2 AND #3	356

Database/Register	Search strategies		Results
World Health Organization International Clinical Trials Registry Platform (WHO ICTRP)	#1	Title: deliver* OR birth OR childbirth OR labor OR labour OR parturition OR obstetric* OR intrapartum	3,042
	#2	Intervention: (perine* OR vulva* OR vagin* OR "genital tract*" OR "reproductive tract*" OR "birth canal" OR “obstetric canal” OR “parturient canal”) AND (“warm compress*” OR “warm application” OR “warm packs” OR warm* OR “heat therapy” OR “heat application” OR heat* OR thermotherapy OR physiotherapy OR "physical therapy")	13
	#3	#1 AND #2	4
Cochrane Central Register of Controlled Trials (CENTRAL)	#1	MeSH descriptor: [Perineum] explode all trees	433
	#2	MeSH descriptor: [Vulva] explode all trees	154
	#3	MeSH descriptor: [Vagina] explode all trees	1,572
	#4	(perine* OR vulva* OR vagin* OR "genital tract*" OR "reproductive tract*" OR "birth canal" OR “obstetric canal” OR “parturient canal”):ti,ab,kw	28,878
	#5	#1 OR #2 OR #3 OR #4	28,893
	#6	(“warm compress*” OR “warm application” OR “warm packs” OR warm* OR “heat therapy” OR “heat application” OR heat* OR thermotherapy OR physiotherapy OR "physical therapy")	45,460
	#7	MeSH descriptor: [Delivery, Obstetric] explode all trees	6,379
	#8	MeSH descriptor: [Labor, Obstetric] explode all trees	2,787
	#9	MeSH descriptor: [Parturition] explode all trees	680
	#10	(deliver* OR birth OR childbirth OR labor OR labour OR parturition OR obstetric* OR intrapartum):ti,ab,kw	139,479
	#11	#7 OR #8 OR #9 OR #10	140,365
	#12	#5 AND #6 AND #11	313
United States National Library of Medicine ClinicalTrials.gov (ClinicalTrials.gov)	#1	Condition/disease: deliver* OR birth OR childbirth OR labor OR labour OR parturition OR obstetric* OR intrapartum	10,152
	#2	Other terms: (perine* OR vulva* OR vagin* OR "genital tract*" OR "reproductive tract*" OR "birth canal" OR “obstetric canal” OR “parturient canal”) AND (“warm compress*” OR “warm packs” OR warm* OR heat* OR thermotherapy OR physiotherapy OR "physical therapy")	115
	#3	#1 AND #2	11
ProQuest Dissertations &Theses Database (PQDT)	#1	SUMMARY= perine* OR vulva* OR vagin* OR "genital tract*" OR "reproductive tract*" OR "birth canal" OR “obstetric canal” OR “parturient canal”	5,946
	#2	SUMMARY= “warm compress*” OR “warm application” OR “warm packs” OR warm* OR “heat therapy” OR “heat application” OR heat* OR thermotherapy OR physiotherapy OR "physical therapy"	149,832
	#3	SUMMARY=deliver* OR birth OR childbirth OR labor OR labour OR parturition OR obstetric* OR intrapartum	240,617
	#4	#1 AND #2 AND #3	35
China Dissertations Database (CDDB)	#1	主题:("会阴") OR 主题:("外阴") OR 主题:("阴道") OR 主题:("产道") OR 主题:("生殖道")	11,784
	#2	主题:("热敷") OR 主题:("热疗") OR 主题:("温敷") OR 主题:("湿敷") OR 主题:("物理疗法") OR 主题:("物理治疗")	3,200
	#3	主题:("分娩") OR 主题:("生产") OR 主题:("产程") OR 主题:("产时") OR 主题:("产中")	672,376
	#4	题名:(剖宫)	816
	#5	(#1 AND #2 AND #3) NOT #4	14

Total results (Search date: from inception to March 15, 2023)

Database/Register	Results
1. PubMed	n=413
2. Embase	n=506
3. CINAHL Plus	n=67
4. Web of Science	n=660
5. China National Knowledge Infrastructure (CNKI)	n=125
6. WanFang Data Knowledge Service Platform (WanFang Data)	n=389
7. Chinese Biomedical Literature Service System (SinoMed)	n=356
8. World Health Organization International Clinical Trials Registry Platform (WHO ICTRP)	n=4

9. Cochrane Central Register of Controlled Trials (CENTRAL)	n=313
10. United States National Library of Medicine ClinicalTrials.gov (ClinicalTrials.gov)	n=11
11. ProQuest Dissertations &Theses Database (PQDT)	n=35
12. China Dissertations Database (CDDB)	n=14
Total	N=2893
