# Supplementary material for: Effects of Perineal Warm Compresses during the Second Stage of Labor on Reducing Perineal Trauma and Relieving Postpartum Perineal Pain in Primiparous Women: A Systematic Review and Meta-Analyses
Source: Healthcare (Basel). 2024 Mar 22;12(7):702. doi: 10.3390/healthcare12070702 (PMC11011582; doi:10.3390/healthcare12070702)
Supplement: Supplementary file 1 [file healthcare-12-00702-s001.zip › Supplementary File S2. The excluded studies with reasons.pdf]

Supplementary File S2. The excluded studies with reasons (N=65)

Reason 1: not RCT (n=20)

[1] WU Yinfen. Effect of Vulvar Hot Packs on Delivery during Second Labor Stage. Nursing Journal of Chinese PLA. 2008, 25(15), 22-23. https://doi.org/10.3969/j.issn.1008-9993.2008.15.012
[2] Walch, A., & Hitchcock, J. (2021). Obstetric anal sphincter injury and implementation of the wha perineal care bundle at a secondary hospital [Conference Abstract]. Australian and New Zealand Journal of Obstetrics and Gynaecology, 61(SUPPL 1), 124. https://doi.org/10.1111/ajo.13345
[3] Aasheim, V., Nilsen, A. B., Lukasse, M., & Reinar, L. M. (2011). Perineal techniques during the second stage of labour for reducing perineal trauma [Review]. Cochrane database of systematic reviews (Online), 12, CD006672. https://www.embase.com/search/results?subaction=viewrecord&id=L560028096&from=export
[4] Aasheim, V., Nilsen, A. B. V., Reinar, L. M., & Lukasse, M. (2017). Perineal techniques during the second stage of labour for reducing perineal trauma [Review]. Cochrane Database of Systematic Reviews, 2017(6). https://doi.org/10.1002/14651858.CD006672.pub3
[5] Arnold, M. J., Sadler, K., & Leli, K. (2021). Obstetric Lacerations: Prevention and Repair [Review]. American Family Physician, 103(12), 745-752. https://www.embase.com/search/results?subaction=viewrecord&id=L635334501&from=export
[6] Bagade, P., & MacKenzie, S. (2010). Outcomes from medium term follow-up of patients with third and fourth degree perineal tears [Article]. Journal of Obstetrics and Gynaecology, 30(6), 609-612. https://doi.org/10.3109/01443615.2010.494205
[7] Elliot, V., Yaskina, M., & Schulz, J. (2019). Obstetrical Anal Sphincter Injuries and the Need for Adequate Care [Article]. Female Pelvic Medicine and Reconstructive Surgery, 25(2), 109-112. https://doi.org/10.1097/SPV.0000000000000684
[8] McGourty, J. (2006). Obstetric risk factors for the development of urinary incontinence after vaginal delivery. Journal of the Association of Chartered Physiotherapists in Women's Health(99), 3-13. https://search.ebscohost.com/login.aspx?direct=true&db=rzh&AN=106203009&lang=zh-cn&site=ehost-live
[9] Shaw-Battista, J., Fineberg, A., Boehler, B., Skubic, B., Woolley, D., & Tilton, Z. (2011). Obstetrician and nurse-midwife collaboration: Successful public health and private practice partnership [Article]. Obstetrics and Gynecology, 118(3), 663-672. https://doi.org/10.1097/AOG.0b013e31822ac86f
[10] Peiris-John, R., Park, C. Y., Wells, S., Kool, B., & Wise, M. R. (2021). Perineal trauma management and follow-up: Are we meeting the standard of care? [Article]. Australian and New Zealand Journal of Obstetrics and Gynaecology, 61(1), 22-29. https://doi.org/10.1111/ajo.13262
[11] Dahlen, H., & Homer, C. (2008). Perineal trauma and postpartum perineal morbidity in Asian and non-Asian primiparous women giving birth in Australia [Article]. JOGNN - Journal of Obstetric, Gynecologic, and Neonatal Nursing, 37(4), 455-463. https://doi.org/10.1111/j.1552-6909.2008.00259.x
[12] Sharley, C. B. (1970). The value of physiotherapy in obstetrics. Med J Aust, 1(23), 1159-1162.
[13] Sleeman, M., McBride, C., & Pill, W. (2021). OASIS injuries in primigravidae women following the introduction of a structured perineal protection bundle. A regional experience [Conference Abstract]. Australian and New Zealand Journal of Obstetrics and Gynaecology, 61(SUPPL 1), 15. https://doi.org/10.1111/ajo.13346
[14] Faraz, S., Vasudevan, V., Ahmed, H. M. A., Varghese, D., Augustine, N., Pillai, U. V., Ammar, A., & Aftab, N. (2022). The Effect of Warm Compress and Proper Perineal Support Technique on Prevention of Severe Perineal Trauma [Article]. Dubai Medical Journal, 5(4), 238-243. https://doi.org/10.1159/000526161
[15] Lei Yan, Yin Minghua, Xiang Huaju, Wang Linyun. Effect of perineal hot compress on perineal pain and injury in the second stage of labor. Chinese and foreign medicine Research. 2018, 16(28), 153-154. https://doi.org/10.14033/j.cnki.cfmr.2018.28.075
[16] Dahlen, H. G., Homer, C. S. E., Cooke, M., Upton, A. M., Nunn, R. A., & Brodrick, B. S. (2009). 'Soothing the ring of fire': Australian women's and midwives' experiences of using perineal warm packs in the second stage of labour [Article]. Midwifery, 25(2), e39-e48. https://doi.org/10.1016/j.midw.2007.08.002
[17] Dalbye, R., & Johannessen, H. H. (2022). Two midwives during the second stage of labour to reduce severe perineal trauma. Lancet, 399(10331), 1203-1205. https://doi.org/10.1016/s0140-6736(22)00381-6
[18] Ibrahim, H., Elgzar, W. T. I., & Hassan, H. E. (2017). Effect of warm compresses versus lubricated massage during the second stage of labor on perineal outcomes among primiparous women. IOSR Journal of Nursing and Health Science, 6(4), 64-76. [retrieved by Google Scholar]
[19] Monem, A. H. A. A., El-Habashy, M. M. I., & Yonis, S. H. M. Effect of Hands-on, Hands-off and Warm Compresses Perineal Techniques during the 2 nd stage of labor on Perineal Outcomes among Primiparae with Vaginal Delivery. [retrieved by Google Scholar]
[20] Sarhan, E. A. E.-R., Qasem, E. A., Gamal, A. M., & Khalil, A. K. (2022). Effect of Warm Compresses Versus Lubricated Massage During the Second Stage of Labor on Perineal Integrity Among Primiparous Women. Menoufia Nursing Journal, 7(1), 111-135. [retrieved by Google Scholar]

Reason 2: ineligible population (n=4)

[1] Hartis, R., Mittal, S., & Lloyd, F. (2018). OASIS: Prevention, treatment and follow-up [Conference Abstract]. BJOG: An International Journal of Obstetrics and Gynaecology, 125, 34. https://doi.org/10.1111/1471-0528.15190
[2] Grohregin, K., Krcmar, M., Ondrova, S., Krofta, L., & Feyereisl, J. (2020). OASIS-4-year ultrasound and clinical follow-up [Conference Abstract]. International Urogynecology Journal, 31(SUPPL 1), S47-S48. https://doi.org/10.1007/s00192-020-04555-3
[3] Albers, L. L., Sedler, K. D., Bedrick, E. J., Teaf, D., & Peralta, P. (2005). Midwifery care measures in the second stage of labor and reduction of genital tract trauma at birth: A randomized trial [Article]. Journal of Midwifery and Women's Health, 50(5), 365-372. https://doi.org/10.1016/j.jmwh.2005.05.012
[4] Gaheen, M., & Abo-Hatab, T. (2021). Effect of Utilizing Perineal Massage, Warm Compresses and Hands on Techniques during the Second Stage of Labor on Perineal Outcomes. Tanta Scientific Nursing Journal, 23, 36-60. https://doi.org/10.21608/tsnj.2021.210228 [retrieved by Google Scholar]

Reason 3: ineligible intervention (n=24)

[1] ClinicalTrials.gov Identifier: NCT02588508. 2015. Effectiveness of Warm Packs, Perineal Massage and Hands Off During Labour in the Perineal Outcomes. https://www.clinicaltrials.gov/ct2/show/NCT02588508
[2] ClinicalTrials.gov Identifier: NCT02582580. 2015. Effects of Antenatal Pelvic Floor Preparation Techniques for Childbirth (EAPFPTC). https://www.clinicaltrials.gov/ct2/show/NCT02582580

[3] McCandlish R, Bowler U, van Asten H, Berridge G, Winter C, Sames L, Garcia J, Renfrew M, Elbourne D. A randomised controlled trial of care of the perineum during second stage of normal labour. Br J Obstet Gynaecol. 1998 Dec;105(12):1262-72. doi: 10.1111/j.1471-0528.1998.tb10004.x.
[4] Irct201108277422N. (2012). The effect of heat and cold therapy on labor pain and outcomes [Trial registry record; Clinical trial protocol]. https://trialsearch.who.int/Trial2.aspx?TrialID=IRCT201108277422N1. https://www.cochranelibrary.com/central/doi/10.1002/central/CN-01867903/full
[5] LI Qinmei, Zhang Jiaojiao, FU Jiyun. The application of comprehensive nursing intervention combined with appropriate protection of perineal delivery in primipara delivery. Today nurse, 2019, 26(5), 66-68. https://d.wanfangdata.com.cn/periodical/ChlQZXJpb2RpY2FsQ0hJTmV3UzIwMjMwMTEyEhFkZGhzLXhzYjIwMTkwNTAyNRoIaGhqMmduazY%3D
[6] ClinicalTrials.gov Identifier: NCT04778631.2021. Impact of Thermotherapy During Childbirth on Postpartum Perineal Pain (PERISAFE). http://clinicaltrials.gov/show/NCT04778631
[7] Irct201407115948N. (2014). Effect of heat therapy and cold therapy on delivery outcome [Trial registry record; Clinical trial protocol]. https://trialsearch.who.int/Trial2.aspx?TrialID=IRCT201407115948N2. https://www.cochranelibrary.com/central/doi/10.1002/central/CN-01811231/full
[8] Nct. (2016). Obstetric Perineal Trauma and Physiotherapy [Trial registry record; Clinical trial protocol]. https://clinicaltrials.gov/show/NCT02682212. https://www.cochranelibrary.com/central/doi/10.1002/central/CN-01555806/full
[9] Nct. (2021). Hands on vs Hands Off for Perineal Laceration [Trial registry record; Clinical trial protocol]. https://clinicaltrials.gov/show/NCT04860102. https://www.cochranelibrary.com/central/doi/10.1002/central/CN-02289395/full
[10] Ganji, Z., Shirvani, M. A., Rezaei-Abhari, F., & Danesh, M. (2013). The effect of intermittent local heat and cold on labor pain and child birth outcome. Iran J Nurs Midwifery Res, 18(4), 298-303.
[11] Taavoni, S., Abdolahian, S., Neisani, L., & Haghani, H. (2015). Three noninvasive interventions for physiologic labour pain management: Use of birth ball, sacrum-perinea heat therapy, and combined use of them during active phase [Conference Abstract]. International Journal of Gynecology and Obstetrics, 131, E217. https://www.embase.com/search/results?subaction=viewrecord&id=L72069409&from=export
[12] Taavoni, S., Abdolahian, S., & Haghani, H. (2011). Sacrum-perinea heat therapy for physiologic labor pain management: A randomized control trial study [Conference Abstract]. Regional Anesthesia and Pain Medicine, 36(5), E199. https://www.embase.com/search/results?subaction=viewrecord&id=L70735787&from=export
[13] Taavoni, S., Abdolahian, S., Neisani, L., & Hamid, H. (2016). Labor pain management: Effect of pelvic tilt by birth ball, sacrumperinea heat therapy, and combined use of them, a randomized controlled trial [Conference Abstract]. European Psychiatry, 33, S503. https://doi.org/10.1016/j.eurpsy.2016.01.1851
[14] Taavoni, S., Sheikhan, F., Abdolahian, S., & Ghavi, F. (2016). Birth ball or heat therapy? A randomized controlled trial to compare the effectiveness of birth ball usage with sacrum-perineal heat therapy in labor pain management [Article]. Complementary therapies in clinical practice, 24, 99-102. https://doi.org/10.1016/j.ctcp.2016.04.001
[15] Behmanesh, F., Pasha, H., & Zeinalzadeh, M. (2009). The Effect of Heat Therapy on Labor Pain Severity and Delivery Outcome in Parturient Women. Iranian Red Crescent Medical Journal, 11(2), 188-192. <Go to ISI>://WOS:000265997900014
[16] Akbarzadeh, M., Vaziri, F., Farahmand, M., Masoudi, Z., Amooee, S., & Zare, N. (2016). The Effect of Warm Compress Bistage Intervention on the Rate of Episiotomy, Perineal Trauma, and Postpartum Pain Intensity in Primiparous Women with Delayed Valsalva Maneuver Referring to the Selected Hospitals of Shiraz University of Medical Sciences in 2012-2013 [Article]. Advances in skin & wound care, 29(2), 79-84. https://doi.org/10.1097/01.ASW.0000476073.96442.91
[17] Didevar, M., Navvabi-Rigi, S. D., & Dadkhah, S. (2022). The Effectiveness of Heat Therapy and Cold Therapy in Labor Pain Intensity in Primiparous Women: A Randomized Controlled Trial. Nursing and Midwifery Studies, 11(3), 171-176. https://doi.org/10.4103/nms.nms_87_21
[18] Farahmand, M., Khooshab, E., Hasanzadeh, F., Amooee, S., & Akbarzadeh, M. (2020). The effect of warm compress Bi-stage on pain strength in labor stages and after delivery [Article]. International Journal of Women's Health and Reproduction Sciences, 8(1), 46-52. https://doi.org/10.15296/ijwhr.2020.06
[19] Kaur, J., Sheoran, P., Kaur, S., & Sarin, J. (2020). Effectiveness of Warm Compression on Lumbo-Sacral Region in Terms of Labour Pain Intensity and Labour Outcomes among Nulliparous: an Interventional Study. J Caring Sci, 9(1), 9-12. https://doi.org/10.34172/jcs.2020.002
[20] Edqvist, M., Dahlen, H. G., Häggsgård, C., Tern, H., Ängeby, K., Teleman, P., Ajne, G., & Rubertsson, C. (2022). The effect of two midwives during the second stage of labour to reduce severe perineal trauma (Oneplus): a multicentre, randomised controlled trial in Sweden [Article]. The Lancet, 399(10331), 1242-1253. https://doi.org/10.1016/S0140-6736(22)00188-X
[21] Fahami, F., Behmanesh, F., Valiani, M., & Ashouri, E. (2011). Effect of heat therapy on pain severity in primigravida women. Iran Journal of Nursing and Midwifery Research, 16(1), 113-116.
[22] Taavoni, S., Abdolahian, S., & Haghani, H. (2013). Effect of sacrum-perineum heat therapy on active phase labor pain and client satisfaction: A randomized, controlled trial study [Article]. Pain Medicine (United States), 14(9), 1301-1306. https://doi.org/10.1111/pme.12161
[23] Sun Xiaojin, Y. B., Liu Xinfeng, Yin Aishun, Zhou Qiaoyi, Yang Ruishan, Zhu Rendi, Zhong Caihong. (2014). The effect of hydropathic and hot compress combined massage to delivery in second stage of labor. Nursing practice and research, 11(5), 73-74,75. https://doi.org/10.3969/j.issn.1672-9676.2014.05.038
[24] Li Yimei, W. X., Xie Ailan. (2017). Effect of perineal hot compress during the second stage of labor on perineal trauma and pain. Maternal and Child Health Care of China, 32(23), 6012-6014. https://doi.org/10. 7620 /zgfybj. j. issn. 1001－4411. 2017. 23. 73

Reason 4: ineligible comparison (n=1)

[1] Nct. (2012). Reduction of Perineal Pain After Vaginal Birth With Black Tea: pilot Randomized Study [Trial registry record; Clinical trial protocol]. https://clinicaltrials.gov/show/NCT01626287. https://www.cochranelibrary.com/central/doi/10.1002/central/CN-01504196/full

Reason 5: conference abstracts/ commentary/letter/ InfoPOEMs: decision support (n=5)

[1] Elliot, V., Yaskina, M., & Schulz, J. A. (2018). Obstetrical anal sphincter injuries and the need for adequate care [Conference Abstract]. Female Pelvic Medicine and Reconstructive Surgery, 24(5), S64. https://doi.org/10.1097/SPV.0000000000000625
[2] Musgrove, H. (1997). Perineal preservation and heat application during second stage of labour [Conference Proceeding]. 10th biennial national conference of the australian college of midwives; 1997 april 16-18; melbourne, australia. https://www.cochranelibrary.com/central/doi/10.1002/central/CN-00757488/full

[3] Rouse, D. J. (2008). Perineal outcomes and maternal comfort related to the application of perineal warm packs in the second stage of labor: A randomized controlled study - Commentary [Note]. Obstetrical and Gynecological Survey, 63(5), 286-287. https://doi.org/10.1097/01.ogx.0000311229.06139.c4
[4] Sloame Cohain, J. (2008). Warm compresses in second stage did not lower the rate of sutured perineums in primiparous women. Birth, 35(2), 167. https://doi.org/10.1111/j.1523-536X.2008.00235.x
[5] Warm packs beneficial in labor. (2008). [Journal article]. Journal of the National Medical Association, 100(3), 348 - . https://www.cochranelibrary.com/central/doi/10.1002/central/CN-01782128/full

Reason 6: inappropriate language type (n=5)

[1] Terré-Rull, C., Beneit-Montesinos, J. V., Gol-Gómez, R., Garriga-Comas, N., Ferrer-Comalat, A., & Salgado-Poveda, I. (2014). Application of perineum heat therapy during partum to reduce injuries that require post-partum stitches [Journal article]. Enfermería clínica, 24(4), 241 - 247. https://doi.org/10.1016/j.enfcli.2014.03.007.
[2] Terré, C., Beneit, J. V., Gol, R., Garriga, N., Salgado, I., & Ferrer, A. (2014). Application of thermotherapy in the perineum to reduce perineal pain during childbirth: randomized clinical trial [Aplicación de termoterapia en el periné para reducir el dolor ensayo clínico a perineal durante el parto: eatorizado]. Matronas Profesion, 15(4), 122-129. https://search.ebscohost.com/login.aspx?direct=true&db=rzh&AN=100802865&lang=zh-cn&site=ehost-live
[3] Sohrabi, M., Ivan bagha, R., & Shirinkam, R. (2012). The effectiveness of physical therapy techniques in the second stage of labor on perineal trauma in nulliparous women referring to the teaching hospital of Emam khomeini--Khalkhal. Journal of Urmia Nursing & Midwifery Faculty, 10(3), 1-7. https://search.ebscohost.com/login.aspx?direct=true&db=rzh&AN=82741964&lang=zh-cn&site=ehost-live
[4] Vaziri, F., Farahmand, M., Samsami, A., Forouhari, S., Hadianfard, M. J., & Sayadi, M. (2014). The effects of warm perineum compress during the second phase of labor on first- birth outcomes. Modern Care. [retrieved by Google Scholar]
[5] Mamuk R, Nimet SG. (2013). Effect of warm application to the perineum in vaginal labor on perineal integrity and pain [Vajinal doğumda perineye sıcak uygulamanın perine bütünlüğü ve ağrıya etkisi]. Journal of Human Sciences, 10(2), 48–66. Retrieved from https://www.j-humansciences.com/ojs/index.php/IJHS/article/view/2551 [retrieved by Google Scholar]

Reason 7: duplicates (n=2)

[1] Nct. (2015). Effectiveness of Warm Packs, Perineal Massage and Hands Off During Labour in the Perineal Outcomes [Trial registry record; Clinical trial protocol]. https://clinicaltrials.gov/show/NCT02588508. https://www.cochranelibrary.com/central/doi/10.1002/central/CN-01493303/full
[2] Edqvist, M., Dahlen, H. G., Häggsgård, C., Tern, H., Ängeby, K., Teleman, P., Ajne, G., & Rubertsson, C. (2022). The Effect of Two Midwives During the Second Stage of Labour to Reduce Severe Perineal Trauma (Oneplus): A Multicentre, Randomized Controlled Trial in Sweden [Note]. Obstetrical and Gynecological Survey, 77(9), 513-515. https://doi.org/10.1097/01.ogx.0000889864.15959.db

Reason 8: clinical registration trials with no available results (n=4)

[1] Irct2015031021020N. (2015). Compared the effect of heat and cold therapy on outcomes of labor [Trial registry record; Clinical trial protocol]. https://trialsearch.who.int/Trial2.aspx?TrialID=IRCT2015031021020N1. https://www.cochranelibrary.com/central/doi/10.1002/central/CN-01815184/full
[2] Irct2014051511706N. (2014). The effect of warm compress bi-stage intervention on prevention of perineal trauma and second stage pain intensity and duration the first stage and second stage in primiparous with delayed valsalva maneuver [Trial registry record; Clinical trial protocol]. https://trialsearch.who.int/Trial2.aspx?TrialID=IRCT2014051511706N7. https://www.cochranelibrary.com/central/doi/10.1002/central/CN-01843509/full
[3] Irct2012111211422N. (2013). Warm compress on the injury severity of perinea pain in labor [Trial registry record; Clinical trial protocol]. https://trialsearch.who.int/Trial2.aspx?TrialID=IRCT2012111211422N1. https://www.cochranelibrary.com/central/doi/10.1002/central/CN-01854350/full
[4] Irct2012072410327N. (2012). Warm compress on the injury severity of perineal pain in labor [Trial registry record; Clinical trial protocol]. https://trialsearch.who.int/Trial2.aspx?TrialID=IRCT2012072410327N2. https://www.cochranelibrary.com/central/doi/10.1002/central/CN-01844477/full
