# Supplementary material for: Effects of Perineal Warm Compresses during the Second Stage of Labor on Reducing Perineal Trauma and Relieving Postpartum Perineal Pain in Primiparous Women: A Systematic Review and Meta-Analyses
Source: Healthcare (Basel). 2024 Mar 22;12(7):702. doi: 10.3390/healthcare12070702 (PMC11011582; doi:10.3390/healthcare12070702)
Supplement: Supplementary file 1 [file healthcare-12-00702-s001.zip › Supplementary File S3. The titles of the included studies.pdf]

Supplementary File S3. The titles of the included studies (N=7)

[1] Dahlen, H. G., Homer, C. S. E., Cooke, M., Upton, A. M., Nunn, R., & Brodrick, B. (2007). Perineal outcomes and maternal comfort related to the application of perineal warm packs in the second stage of labor: A randomized controlled trial [Article]. Birth, 34(4), 282-290. https://doi.org/10.1111/j.1523-536X.2007.00186.x

[2] Ahmad ER, Turky HA. Effect of applying warm perineal packs during the second stage of labor on perineal pain among primiparous women. Al-Azhar Assiut Med J. 2010;8(3):1–26. [retrieved by Google Scholar]

[3] Essa, R. M., & Ismail, N. I. A. A. . (2015). Effect of second stage perineal warm compresses on perineal pain and outcome among primiparae. Journal of Nursing Education and Practice, 6(4). https://doi.org/10.5430/jnep.v6n4p48 [retrieved by Google Scholar]

[4] Alihosseni, F., Abedi, P., Afshary, P., Haghighi, M. R., & Hazeghi, N. (2018). Investigating the effect of perineal heating pad on the frequency of episiotomies and perineal tears in primiparous females. Medical-Surgical Nursing Journal, 7(1). https://doi.org/10.5812/msnj.82588 [retrieved by Google Scholar]

[5] Modoor, S., Fouly, H., & Rawas, H. (2021). The effect of warm compresses on perinea! tear and pain intensity during the second stage of labor: A randomized controlled trial. Belitung Nursing Journal, 7(3), 210-218. https://doi.org/10.33546/bnj.1452

[6] Türkmen, H., Çetinkaya, S., Apay, E., Karamüftüoğlu, D., & Klllç, H. (2021). The Effect of Perineal Warm Application on Perineal Pain, Perineal Integrity, and Postpartum Comfort in the Second Stage of Labor: Randomized Clinical Trial [Article]. Complementary Medicine Research, 28(1), 23-30. https://doi.org/10.1159/000507605

[7] Liao Yumei. (2021). Observation on the Effect of Perineal Hot Compress in the Second Stage of Labor onPerineal Pain and Injury. World Latest Medicine Information, 21(96), 154-155. https://doi.org/10.3969/j.issn.1671-3141.2021.96.052

The list of contacting the original authors for clarification or data provision

[1] Modoor, S., Fouly, H., & Rawas, H. (2021). The effect of warm compresses on perinea! tear and pain intensity during the second stage of labor: A randomized controlled trial. Belitung Nursing Journal, 7(3), 210-218. https://doi.org/10.33546/bnj.1452. Emai: foulyh@ksau-hs.edu.sa; holda.elfouly2@aun.edu.eg

[2] Essa, R. M., & Ismail, N. I. A. A. (2015). Effect of second stage perineal warm compresses on perineal pain and outcome among primiparae. Journal of Nursing Education and Practice, 6(4). https://doi.org/10.5430/jnep.v6n4p48. Email: rashaessa111@yahoo.com.
