# Supplementary material for: Effects of Perineal Warm Compresses during the Second Stage of Labor on Reducing Perineal Trauma and Relieving Postpartum Perineal Pain in Primiparous Women: A Systematic Review and Meta-Analyses
Source: Healthcare (Basel). 2024 Mar 22;12(7):702. doi: 10.3390/healthcare12070702 (PMC11011582; doi:10.3390/healthcare12070702)
Supplement: Supplementary file 1 [file healthcare-12-00702-s001.zip › Table S2. Data extraction of maternal outcomes.pdf]

Table S2.1. Primary maternal outcomes (N=7).

	Dahlen et al, 2007	Ahmad et al, 2010	Essa et al, 2015	Alihosseni et al, 2018	Modoor et al, 2021	Türkmen et al, 2021	Liao, 2021	Total (warm compresses vs standard care)	RR or MD (95% CI)
Intact perineum<br>OIS: 186	77/360(21.39%) vs 73/357(20.45%)	NR	50/80(62.50%) vs 2/80 (2.50%)	14/54(25.93%) vs 8/53 (15.09%)	11/50(22.00%) vs 5/50(10.00%)	8/50(16.00%) vs 1/50(2.00%)	3/39(7.69%) vs 0/39(0.00%)	163/633(25.75%) vs 89/629(14.15%)	3.36 (1.22 to 9.27)
Perineum lacerations<br>OIS: 406	15/360(4.17%) vs 31/357(8.68%)	NR	2/80(2.5%) vs 25/80(31.25%)	21/54(38.89%) vs 22/53(41.51%)	31/50(62.00%) vs 41/50(82.00%)	3/50(6.00%) vs 5/50(10.00%)	36/39(92.30%) vs 39/39(100%)	108/633(17.06%) vs 163/629(25.91%)	0.66 (0.54 to 0.82)
First-degree<br>OIS:521	NR	NR	2/80(2.50%) vs 0/80 (0.00%)	14/54(25.93%) vs 13/53 (24.53%)	19/50(38.00%) vs 13/50(26.00%)	3/50(6.00%) vs 5/50(10.00%)	27/39 (69.23%) vs 14/39 (35.90%)	65/273(23.81%) vs 45/272(16.54%)	1.43 (1.05 to 1.95)
Second-degree<br>OIS:70	NR	NR	0/80(0.00%) vs 9/80 (11.25%)	7/54(12.96%) vs 9/53(16.98%)	10/50(20.00%) vs 24/50(48.00%)	NR	9/39 (23.08%) vs 24/39 (61.54%)	26/223(11.16%) vs 66/222(29.73%)	0.40 (0.27 to 0.59)
Third- and/or fourth-degree<br>OIS:215	15/360(4.17%) vs 31/357(8.68%)	NR	0/80(0.00%) vs 16/80 (2.00%)	NR	2/50(4.00%) vs 4/50(8.00%)	NR	0/39 (0.0%) vs 1/39 (2.56%)	17/529(3.21%) vs 52/526(9.89%)	0.34 (0.20 to 0.57)
Perineal trauma requiring suture<br>OIS: 161	283/360(78.61%) vs 284/357(79.55%)	NR	26/80(3.25%) vs 78/80 (97.50%)	NR	NR	41/50(82.00%) vs 49/50(98.00%)	NR	350/490(71.43%) vs 411/487(84.39%)	0.68 (0.45 to 1.02)
Episiotomy<br>OIS:358	39/360(10.83%) vs 41/357(11.48%)	NR	26/80(32.50%) vs 50/80 (62.50%)	11/54(20.37%) vs 22/53 (41.51%)	9/50(18.00%) vs 12/50(24.00%)	35/50(70.00%) vs 48/50(96.00%)	NR	120/594(20.20%) vs 173/590(29.32%)	0.69 (0.58 to 0.83)
Perineal pain									

	Dahlen et al, 2007	Ahmad et al, 2010	Essa et al, 2015	Alihosseni et al, 2018	Modoor et al, 2021	Türkmen et al, 2021	Liao, 2021	Total (warm compresses vs standard care)	RR or MD (95% CI)
Immediately after delivery	NR	5.97±1.49 vs 7.89±1.91 50/50	*-Mild 1-3: 20/80 vs 0/80<br>- Moderate 4-6: 52/80 vs 16/80<br>- Severe 7-9: 8/80 vs 52/80<br>- Unbearable ≥ 10: 0/80 vs 12/80	NR	*-Mild: 20/50 vs 26/50<br>-Moderate: 26/50 vs 17/50<br>-Severe: 4/50 vs 7/50	2.20±1.72 vs 3.64±2.07 50/50	NR	NR	-1.71 (-2.20 to -1.21)
The first day after delivery	3.86±2.3 vs 4.67 ±2.3	4.82±1.59 vs 7.58±1.57	NR	NR	NR	NR	3.05±0.93 vs 3.56±1.13	NR	-1.04 (-1.29 to -0.79)
The second day after delivery	3.00±2.1 vs 3.7± 2.2	NR	NR	NR	NR	NR	2.41±0.78 vs 2.94±1.10	NR	-0.64 (-0.89 to -0.39)

* The pain scores in these two studies could not be converted because we could not obtain the relevant original data.

Table S2.2. Secondary maternal outcomes (N=7).

	Dahlen et al, 2007	Ahmad et al, 2010	Essa et al, 2015	Alihosseni et al, 2018	Modoor et al, 2021	Türkmen et al, 2021	Liao, 2021
Perineal pain							
Before intervention	NR	8.88±1.13 vs 7.89±1.91	NR	NR	NR	9.94±0.42 vs 9.86±0.57	NR
During the delivery	NR	NR	NR	NR	NR	8.54±1.38 vs 9.86±0.57	NR

	Dahlen et al, 2007	Ahmad et al, 2010	Essa et al, 2015	Alihosseni et al, 2018	Modoor et al, 2021	Türkmen et al, 2021	Liao, 2021
2 hours after delivery	NR	NR	NR	NR	NR	0.30±0.78 vs 0.68±0.98	NR
Need for pain relief during the second stage	NR	NR	6/80(7.50%) vs 36/80 (45.00%)	NR	NR	NR	NR
Perineal swelling							
First-degree	NR	NR	NR	NR	NR	NR	22/39 (56.41%) vs 14/39 (35.90%)
Second-degree	NR	NR	NR	NR	NR	NR	10/39 (25.64%) vs 20/39 (51.28%)
Third-degree	NR	NR	NR	NR	NR	NR	6/39 (15.38%) vs 8/39 (20.51%)
Fourth-degree	NR	NR	NR	NR	NR	NR	1/39 (2.56%) vs 7/39 (17.95%)
Postpartum comfort level	NR	NR	NR	NR	NR	4.13±0.31 vs 4.09±0.31	NR
Urinary incontinence postpartum							
At 6 weeks	36/276(13.04%) vs 46/277(16.61%)	NR	NR	NR	NR	NR	NR
At 3 months	26/277(9.39%) vs 59/262(22.52%)	NR	NR	NR	NR	NR	NR
