# Supplementary material for: Effects of Perineal Warm Compresses during the Second Stage of Labor on Reducing Perineal Trauma and Relieving Postpartum Perineal Pain in Primiparous Women: A Systematic Review and Meta-Analyses
Source: Healthcare (Basel). 2024 Mar 22;12(7):702. doi: 10.3390/healthcare12070702 (PMC11011582; doi:10.3390/healthcare12070702)
Supplement: Supplementary file 1 [file healthcare-12-00702-s001.zip › Table S3. Summary of risk of bias assessment.pdf]

Table S3. Summary of risk of bias assessment

Included Study	Assessors	Risk of Bias																											Overall Bias
		Bias Arising from the Randomization Process				Bias Due to Deviations from Intended Interventions								Bias Due to Missing Outcome Data					Bias in Measurement of the Outcome						Bias in Selection of the Reported Result				
		Q 1.1	Q 1.2	Q 1.3	D1	Q 2.1	Q 2.2	Q 2.3	Q 2.4	Q 2.5	Q 2.6	Q 2.7	D2	Q 3.1	Q 3.2	Q 3.3	Q 3.4	D3	Q 4.1	Q 4.2	Q 4.3	Q 4.4	Q 4.5	D4	Q 5.1	Q 5.2	Q 5.3	D5	
01 Dahlen et al, 2007	1	Y	Y	N	Low	Y	Y	N	NA	NA	PY	NA	Low	N	PN	PN	NA	Low	N	N	N	NA	NA	Low	PY	N	N	Low	Low
	2	Y	Y	N	Low	Y	Y	N	NA	NA	Y	NA	Low	N	PN	PN	NA	Low	N	N	N	NA	NA	Low	Y	N	N	Low	Low
	Final Result	Y	Y	N	Low	Y	Y	N	NA	NA	PY	NA	Low	N	PN	PN	NA	Low	N	N	N	NA	NA	Low	PY	N	N	Low	Low
02 Ahmad et al, 2010	1	PY	NI	PN	Some concerns	PY	Y	PN	NA	NA	Y	NA	Low	Y	NA	NA	NA	Low	N	N	Y	PN	NA	Low	Y	N	N	Low	Some concerns
	2	PY	NI	PN	Some concerns	Y	Y	N	NA	NA	PY	NA	Low	Y	NA	NA	NA	Low	N	N	PY	PN	NA	Low	Y	N	N	Low	Some concerns
	Final Result	PY	NI	PN	Some concerns	PY	Y	PN	NA	NA	PY	NA	Low	Y	NA	NA	NA	Low	N	N	PY	PN	NA	Low	Y	N	N	Low	Some concerns
3 Essa et al, 2015	1	PN	Y	PN	Some concerns	PY	Y	PN	NA	NA	PY	NA	Low	Y	NA	NA	NA	Low	PN	N	Y	PN	NA	Low	PY	PN	PN	Low	Some concerns
	2	PN	PY	PN	Some concerns	PY	Y	N	NA	NA	PY	NA	Low	Y	NA	NA	NA	Low	PN	PN	PY	PN	NA	Low	Y	PN	PN	Low	Some concerns
	Final Result	PN	Y	PN	Some concerns	PY	Y	PN	NA	NA	PY	NA	Low	Y	NA	NA	NA	Low	PN	N	Y	PN	NA	Low	PY	PN	PN	Low	Some concerns
4 Alihosseni et al, 2018	1	NI	NI	PN	Some concerns	N	Y	PN	NA	NA	PY	NA	Low	Y	NA	NA	NA	Low	N	PN	Y	PN	NA	Low	PY	PN	PN	Low	Some concerns
	2	NI	NI	N	Some concerns	N	Y	PN	NA	NA	PY	NA	Low	N	PN	PN	NA	Low	N	PN	Y	PN	NA	Low	PY	PN	PN	Low	Some concerns
	Final Result	NI	NI	PN	Some concerns	N	PY	PN	NA	NA	PY	NA	Low	N	N	PN	NA	Low	N	N	PY	PN	NA	Low	PY	N	N	Low	Some concerns
05 Modoor et al, 2021	1	Y	Y	N	Low	Y	PY	PN	NA	NA	Y	NA	Low	Y	NA	NA	NA	Low	N	N	Y	N	NA	Low	Y	N	N	Low	Low
	2	Y	Y	N	Low	Y	Y	N	NA	NA	Y	NA	Low	Y	NA	NA	NA	Low	N	N	PY	PN	NA	Low	Y	N	N	Low	Low
	Final Result	Y	Y	N	Low	Y	PY	PN	NA	NA	Y	NA	Low	Y	NA	NA	NA	Low	N	N	PY	PN	NA	Low	Y	N	N	Low	Low
06 Türkmen et al, 2021	1	Y	Y	N	Low	Y	Y	N	NA	NA	Y	NA	Low	Y	NA	NA	NA	Low	N	N	Y	PN	NA	Low	Y	N	N	Low	Low
	2	Y	Y	N	Low	Y	Y	N	NA	NA	Y	NA	Low	Y	NA	NA	NA	Low	N	N	PY	PN	NA	Low	Y	N	N	Low	Low
	Final Result	Y	Y	N	Low	Y	Y	N	NA	NA	Y	NA	Low	Y	NA	NA	NA	Low	N	N	PY	PN	NA	Low	Y	N	N	Low	Low
07 Liao 2021	1	Y	NI	N	Some concerns	PY	PY	PN	NA	NA	PY	NA	Low	Y	NA	NA	NA	Low	N	PN	Y	PY	PN	Some concerns	PY	PN	PN	Low	Some concerns
	2	Y	NI	N	Some concerns	PY	PY	PN	NA	NA	PY	NA	Low	Y	NA	NA	NA	Low	N	N	Y	PY	PN	Some concerns	Y	N	N	Low	Some concerns
	Final Result	Y	NI	N	Some concerns	PY	PY	PN	NA	NA	PY	NA	Low	Y	NA	NA	NA	Low	N	PN	Y	PY	PN	Some concerns	PY	PN	PN	Low	Some concerns

Notes: Yes (Y), Probably Yes (PY), Probably No (PN), No (N), No Information (NI) or Not Applicable (NA); Low risk of bias (Low), Some concerns or High risk of bias (High).

Supplementary Table 4 Summary of the results of sensitivity analyses

Outcomes	Subgroups	Removed study	Statistical results
1. Intact perineum	/	Alihosseni et al, 2018	RR: 4.37 [1.04, 18.38], *P<0.00001*, I^2=87%
		Dahlen et al, 2007	RR: 4.86 [1.44, 16.35], *P*=0.003, I^2=75%
		Essa et al, 2015	RR: 1.73 [0.95, 3.16], *P*=0.09, I^2=50%
		Liao, 2021	RR: 3.16 [1.09, 9.17], *P<0.00001*, I^2=87%
		Modoor et al, 2021	RR: 3.94 [1.09, 14.25], *P<0.00001*, I^2=87%
		Türkmen et al, 2021	RR: 2.97 [1.02, 8.64], *P<0.00001*, I^2=86%
2. Perineal lacerations	Subgroup 1: first-degree	Alihosseni et al, 2018	RR: 1.58 [1.11, 2.25], *P*=0.36, I^2=7%
		Essa et al, 2015	RR: 1.39 [1.02, 1.90], *P*=0.26, I^2=25%
		Liao, 2021	RR: 1.21 [0.81, 1.83], *P*=0.49, I^2=0%
		Modoor et al, 2021	RR: 1.42 [0.99, 2.05], *P*=0.20, I^2=35%
		Türkmen et al, 2021	RR: 1.54 [1.21, 2.11], *P*=0.43, I^2=0%
	Subgroup 2: second-degree	Alihosseni et al, 2018	RR: 0.34 [0.22, 0.53], *P*=0.34, I^2=8%
		Essa et al, 2015	RR: 0.45 [0.31, 0.67], *P*=0.43, I^2=0%
		Liao, 2021	RR: 0.41 [0.25, 0.67], *P*=0.15, I^2=48%
		Modoor et al, 2021	RR: 0.39 [0.23, 0.64], *P*=0.13, I^2=51%
	Subgroup 3: third- and/or fourth-degree	Dahlen et al, 2007	Peto OR: 0.17 [0.07, 0.39], *P*=0.31, I^2=14%
		Essa et al, 2015	Peto OR: 0.46 [0.27, 0.81], *P*=0.82, I^2=0%
		Liao, 2021	Peto OR: 0.34 [0.21, 0.56], *P*=0.05, I^2=67%
		Modoor et al, 2021	Peto OR: 0.32 [0.19, 0.54], *P*=0.05, I^2=66%
3. Perineal lacerations requiring suture	/	Dahlen et al, 2007	RR: 0.53 [0.15, 1.85], *P<0.00001*, I^2=98%
		Essa et al, 2015	RR: 0.92 [0.78, 1.08], *P*=0.03, I^2=78%
		Türkmen et al, 2021	RR: 0.58 [0.18, 1.82], *P<0.00001*, I^2=98%
4. Incidence of episiotomy	/	Alihosseni et al, 2018	RR: 0.72 [0.60, 0.87], *P*=0.18, I^2=38%
		Dahlen et al, 2007	RR: 0.61 [0.51, 0.74], *P*=0.19, I^2=38%
		Essa et al, 2015	RR: 0.76 [0.62, 0.93], *P*=0.37, I^2=5%
		Modoor et al, 2021	RR: 0.69 [0.57, 0.82], *P*=0.11, I^2=51%
		Türkmen et al, 2021	RR: 0.68 [0.53, 0.86], *P*=0.13, I^2=47%
5. Postpartum perineal pain	Subgroup 1: immediately after delivery	Ahmad et al, 2010	MD: -1.44 [-2.19, -0.69], *P*=0.0002
		Türkmen et al, 2021	MD: -1.92 [-2.59, -1.25], *P<0.00001*
	Subgroup 2: the first day after delivery	Ahmad et al, 2010	MD: -0.70 [-0.98, -0.42], *P*=0.30, I^2=6%
		Dahlen et al, 2007	MD: -1.63 [-3.83, -0.58], *P<0.00001*, I^2=97%
		Liao, 2021	MD: -1.77 [-3.68, -0.14], *P<0.00001*, I^2=97%
	Subgroup 3: the second day after delivery	Dahlen et al, 2007	MD: -0.53 [-0.95, -0.11], *P*=0.01
		Liao, 2021	MD: -0.70 [-1.01, -0.39], *P<0.0001*

Supplementary Table 5 GRADE evidence profile of maternal outcomes

Certainty assessment							№ of patients		Effect		Certainty	Importance
№ of studies	Study design	Risk of bias	Inconsistency	Indirectness	Imprecision	Other considerations	warm compresses	standard care	Relative (95% CI)	Absolute (95% CI)		
**Intact perineum**												

Certainty assessment							№ of patients		Effect		Certainty	Importance
№ of studies	Study design	Risk of bias	Inconsistency	Indirectness	Imprecision	Other considerations	warm compresses	standard care	Relative (95% CI)	Absolute (95% CI)		
6	randomised trials	not serious	serious	not serious	not serious	publication bias strongly suspected	163/633 (25.8%)	89/629 (14.1%)	**RR 3.36** (1.22 to 9.27)	**334 more per 1,000** (from 31 more to 1,000 more)	⊕⊕◯◯ Low	-
**Perineal lacerations**												
6	randomised trials	not serious	serious	not serious	not serious	publication bias strongly suspected	108/1025 (10.5%)	163/1020 (16.0%)	**RR 0.66** (0.54 to 0.82)	**54 fewer per 1,000** (from 74 fewer to 29 fewer)	⊕⊕◯◯ Low	-
**Perineal lacerations - First-degree perineal lacerations**												
5	randomised trials	serious	not serious	not serious	serious	publication bias strongly suspected	65/273 (23.8%)	45/272 (16.5%)	**RR 1.43** (1.05 to 1.95)	**71 more per 1,000** (from 8 more to 157 more)	⊕◯◯◯ Very low	-
**Perineal lacerations - Second-degree perineal lacerations**												
4	randomised trials	serious	not serious	not serious	not serious	publication bias strongly suspected	26/223 (11.7%)	66/222 (29.7%)	**RR 0.40** (0.27 to 0.59)	**178 fewer per 1,000** (from 217 fewer to 122 fewer)	⊕⊕◯◯ Low	-
**Perineal lacerations - Third- and/or fourth-degree perineal lacerations**												
4	randomised trials	not serious	not serious	not serious	not serious	publication bias strongly suspected	17/529 (3.2%)	52/526 (9.9%)	**RR 0.34** (0.20 to 0.57)	**65 fewer per 1,000** (from 79 fewer to 43 fewer)	⊕⊕⊕◯ Moderate	-
**Perineal lacerations requiring suture**												

Certainty assessment							№ of patients		Effect		Certainty	Importance
№ of studies	Study design	Risk of bias	Inconsistency	Indirectness	Imprecision	Other considerations	warm compresses	standard care	Relative (95% CI)	Absolute (95% CI)		
3	randomised trials	not serious	serious	not serious	not serious	publication bias strongly suspected	350/490 (71.4%)	411/487 (84.4%)	**RR 0.68** (0.45 to 1.02)	**270 fewer per 1,000** (from 464 fewer to 17 more)	⨁⨁◯◯ Low	-
**Episiotomy**												
5	randomised trials	not serious	not serious	not serious	not serious	publication bias strongly suspected	120/594 (20.2%)	173/590 (29.3%)	**RR 0.69** (0.58 to 0.83)	**91 fewer per 1,000** (from 123 fewer to 50 fewer)	⨁⨁⨁◯ Moderate	-
**Postpartum perineal pain**												
4	randomised trials	not serious	serious	not serious	not serious	publication bias strongly suspected	948	942	-	MD **0.94 lower** (1.1 lower to 0.77 lower)	⨁⨁◯◯ Low	-
**Postpartum perineal pain - Immediately after delivery**												
2	randomised trials	serious	not serious	not serious	serious	publication bias strongly suspected	100	100	-	MD **1.71 lower** (2.2 lower to 1.21 lower)	⨁◯◯◯ Very low	-
**Postpartum perineal pain - The first day after delivery**												
3	randomised trials	not serious	serious	not serious	not serious	publication bias strongly suspected	449	446	-	MD **1.04 lower** (1.29 lower to 0.79 lower)	⨁⨁◯◯ Low	-
**Postpartum perineal pain - The second day after delivery**												

Certainty assessment							№ of patients		Effect		Certainty	Importance
№ of studies	Study design	Risk of bias	Inconsistency	Indirectness	Imprecision	Other considerations	warm compresses	standard care	Relative (95% CI)	Absolute (95% CI)		
2	randomised trials	not serious	not serious	not serious	not serious	publication bias strongly suspected	399	396	-	MD **0.64 lower** (0.89 lower to 0.39 lower)	⊕⊕⊕◯ Moderate	-

Notes: ①CI: confidence interval, ②MD: mean difference, ③RR: risk ratio.

Supplementary Figure 1 Forest plot of the effect of perineal warm compresses during childbirth on intact perineum
