# Supplementary material for: Effects of Perineal Warm Compresses during the Second Stage of Labor on Reducing Perineal Trauma and Relieving Postpartum Perineal Pain in Primiparous Women: A Systematic Review and Meta-Analyses
Source: Healthcare (Basel). 2024 Mar 22;12(7):702. doi: 10.3390/healthcare12070702 (PMC11011582; doi:10.3390/healthcare12070702)
Supplement: Supplementary file 1 [file healthcare-12-00702-s001.zip › Table S4. Summary of the results of sensitivity analyses.pdf]

Table S4. Summary of the results of sensitivity analyses

Outcomes	Subgroups	Removed study	Statistical results
1. Intact perineum	/	Alihosseni et al, 2018	RR: 4.37 [1.04, 18.38], *P<0.00001*, I^2=87%
		Dahlen et al, 2007	RR: 4.86 [1.44, 16.35], *P*=0.003, I^2=75%
		Essa et al, 2015	RR: 1.73 [0.95, 3.16], *P*=0.09, I^2=50%
		Liao, 2021	RR: 3.16 [1.09, 9.17], *P<0.00001*, I^2=87%
		Modoor et al, 2021	RR: 3.94 [1.09, 14.25], *P<0.00001*, I^2=87%
		Türkmen et al, 2021	RR: 2.97 [1.02, 8.64], *P<0.00001*, I^2=86%
2. Perineal lacerations	Subgroup 1: first-degree	Alihosseni et al, 2018	RR: 1.58 [1.11, 2.25], *P*=0.36, I^2=7%
		Essa et al, 2015	RR: 1.39 [1.02, 1.90], *P*=0.26, I^2=25%
		Liao, 2021	RR: 1.21 [0.81, 1.83], *P*=0.49, I^2=0%
		Modoor et al, 2021	RR: 1.42 [0.99, 2.05], *P*=0.20, I^2=35%
		Türkmen et al, 2021	RR: 1.54 [1.21, 2.11], *P*=0.43, I^2=0%
	Subgroup 2: second-degree	Alihosseni et al, 2018	RR: 0.34 [0.22, 0.53], *P*=0.34, I^2=8%
		Essa et al, 2015	RR: 0.45 [0.31, 0.67], *P*=0.43, I^2=0%
		Liao, 2021	RR: 0.41 [0.25, 0.67], *P*=0.15, I^2=48%
		Modoor et al, 2021	RR: 0.39 [0.23, 0.64], *P*=0.13, I^2=51%
	Subgroup 3: third- and/or fourth-degree	Dahlen et al, 2007	Peto OR: 0.17 [0.07, 0.39], *P*=0.31, I^2=14%
		Essa et al, 2015	Peto OR: 0.46 [0.27, 0.81], *P*=0.82, I^2=0%
		Liao, 2021	Peto OR: 0.34 [0.21, 0.56], *P*=0.05, I^2=67%
		Modoor et al, 2021	Peto OR: 0.32 [0.19, 0.54], *P*=0.05, I^2=66%
3. Perineal lacerations requiring suture	/	Dahlen et al, 2007	RR: 0.53 [0.15, 1.85], *P<0.00001*, I^2=98%
		Essa et al, 2015	RR: 0.92 [0.78, 1.08], *P*=0.03, I^2=78%
		Türkmen et al, 2021	RR: 0.58 [0.18, 1.82], *P<0.00001*, I^2=98%
4. Incidence of episiotomy	/	Alihosseni et al, 2018	RR: 0.72 [0.60, 0.87], *P*=0.18, I^2=38%
		Dahlen et al, 2007	RR: 0.61 [0.51, 0.74], *P*=0.19, I^2=38%
		Essa et al, 2015	RR: 0.76 [0.62, 0.93], *P*=0.37, I^2=5%
		Modoor et al, 2021	RR: 0.69 [0.57, 0.82], *P*=0.11, I^2=51%
		Türkmen et al, 2021	RR: 0.68 [0.53, 0.86], *P*=0.13, I^2=47%
5. Postpartum perineal pain	Subgroup 1: immediately after delivery	Ahmad et al, 2010	MD: -1.44 [-2.19, -0.69], *P*=0.0002
		Türkmen et al, 2021	MD: -1.92 [-2.59, -1.25], *P<0.00001*
	Subgroup 2: the first day after delivery	Ahmad et al, 2010	MD: -0.70 [-0.98, -0.42], *P*=0.30, I^2=6%
		Dahlen et al, 2007	MD: -1.63 [-3.83, -0.58], *P<0.00001*, I^2=97%
		Liao, 2021	MD: -1.77 [-3.68, -0.14], *P<0.00001*, I^2=97%
	Subgroup 3: the second day after delivery	Dahlen et al, 2007	MD: -0.53 [-0.95, -0.11], *P*=0.01
		Liao, 2021	MD: -0.70 [-1.01, -0.39], *P<0.0001*
