# Supplementary material for: Effects of Perineal Warm Compresses during the Second Stage of Labor on Reducing Perineal Trauma and Relieving Postpartum Perineal Pain in Primiparous Women: A Systematic Review and Meta-Analyses
Source: Healthcare (Basel). 2024 Mar 22;12(7):702. doi: 10.3390/healthcare12070702 (PMC11011582; doi:10.3390/healthcare12070702)
Supplement: Supplementary file 1 [file healthcare-12-00702-s001.zip › Table S5. GRADE evidence profile of maternal outcomes.pdf]

Table S5. GRADE evidence profile of maternal outcomes

Certainty assessment							№ of patients		Effect		Certainty	Importance
№ of studies	Study design	Risk of bias	Inconsistency	Indirectness	Imprecision	Other considerations	warm compresses	standard care	Relative (95% CI)	Absolute (95% CI)		
**Intact perineum**												
6	randomised trials	not serious	serious	not serious	not serious	publication bias strongly suspected	163/633 (25.8%)	89/629 (14.1%)	**RR 3.36** (1.22 to 9.27)	**334 more per 1,000** (from 31 more to 1,000 more)	⊕⊕◯◯ Low	-
**Perineal lacerations**												
6	randomised trials	not serious	serious	not serious	not serious	publication bias strongly suspected	108/1025 (10.5%)	163/1020 (16.0%)	**RR 0.66** (0.54 to 0.82)	**54 fewer per 1,000** (from 74 fewer to 29 fewer)	⊕⊕◯◯ Low	-
**Perineal lacerations - First-degree perineal lacerations**												
5	randomised trials	serious	not serious	not serious	serious	publication bias strongly suspected	65/273 (23.8%)	45/272 (16.5%)	**RR 1.43** (1.05 to 1.95)	**71 more per 1,000** (from 8 more to 157 more)	⊕◯◯◯ Very low	-
**Perineal lacerations - Second-degree perineal lacerations**												
4	randomised trials	serious	not serious	not serious	not serious	publication bias strongly suspected	26/223 (11.7%)	66/222 (29.7%)	**RR 0.40** (0.27 to 0.59)	**178 fewer per 1,000** (from 217 fewer to 122 fewer)	⊕⊕◯◯ Low	-
**Perineal lacerations - Third- and/or fourth-degree perineal lacerations**												
4	randomised trials	not serious	not serious	not serious	not serious	publication bias strongly suspected	17/529 (3.2%)	52/526 (9.9%)	**RR 0.34** (0.20 to 0.57)	**65 fewer per 1,000** (from 79 fewer to 43 fewer)	⊕⊕⊕◯ Moderate	-
**Perineal lacerations requiring suture**												

Certainty assessment							№ of patients		Effect		Certainty	Importance
№ of studies	Study design	Risk of bias	Inconsistency	Indirectness	Imprecision	Other considerations	warm compresses	standard care	Relative (95% CI)	Absolute (95% CI)		
3	randomised trials	not serious	serious	not serious	not serious	publication bias strongly suspected	350/490 (71.4%)	411/487 (84.4%)	**RR 0.68** (0.45 to 1.02)	**270 fewer per 1,000** (from 464 fewer to 17 more)	⨁⨁◯◯ Low	-
**Episiotomy**												
5	randomised trials	not serious	not serious	not serious	not serious	publication bias strongly suspected	120/594 (20.2%)	173/590 (29.3%)	**RR 0.69** (0.58 to 0.83)	**91 fewer per 1,000** (from 123 fewer to 50 fewer)	⨁⨁⨁◯ Moderate	-
**Postpartum perineal pain**												
4	randomised trials	not serious	serious	not serious	not serious	publication bias strongly suspected	948	942	-	MD **0.94 lower** (1.1 lower to 0.77 lower)	⨁⨁◯◯ Low	-
**Postpartum perineal pain - Immediately after delivery**												
2	randomised trials	serious	not serious	not serious	serious	publication bias strongly suspected	100	100	-	MD **1.71 lower** (2.2 lower to 1.21 lower)	⨁◯◯◯ Very low	-
**Postpartum perineal pain - The first day after delivery**												
3	randomised trials	not serious	serious	not serious	not serious	publication bias strongly suspected	449	446	-	MD **1.04 lower** (1.29 lower to 0.79 lower)	⨁⨁◯◯ Low	-
**Postpartum perineal pain - The second day after delivery**												

Certainty assessment							№ of patients		Effect		Certainty	Importance
№ of studies	Study design	Risk of bias	Inconsistency	Indirectness	Imprecision	Other considerations	warm compresses	standard care	Relative (95% CI)	Absolute (95% CI)		
2	randomised trials	not serious	not serious	not serious	not serious	publication bias strongly suspected	399	396	-	MD **0.64 lower** (0.89 lower to 0.39 lower)	⨁⨁⨁◯ Moderate	-

Notes: ①CI: confidence interval, ②MD: mean difference, ③RR: risk ratio.
