# Supplementary figures and images for: Effects of Perineal Warm Compresses during the Second Stage of Labor on Reducing Perineal Trauma and Relieving Postpartum Perineal Pain in Primiparous Women: A Systematic Review and Meta-Analyses
Source: Healthcare (Basel). 2024 Mar 22;12(7):702. doi: 10.3390/healthcare12070702 (PMC11011582; doi:10.3390/healthcare12070702)

Figure S1. Effect of warm compresses on intact perineum

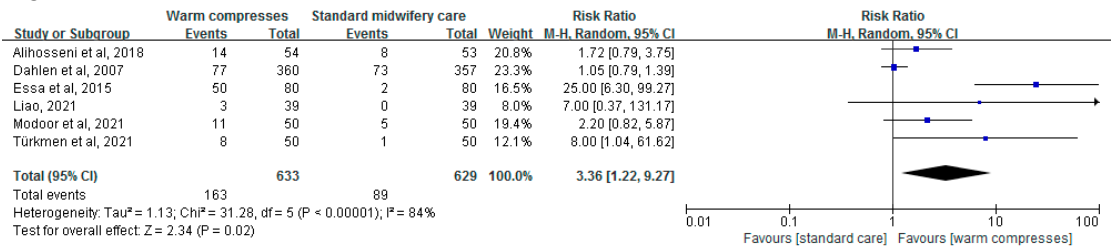

Supplement: Supplementary file 1 [file healthcare-12-00702-s001.zip › Figure S1. Effect of warm compresses on intact perineum.pdf]

Figure S2. Effect of warm compresses on perineal lacerations requiring suture

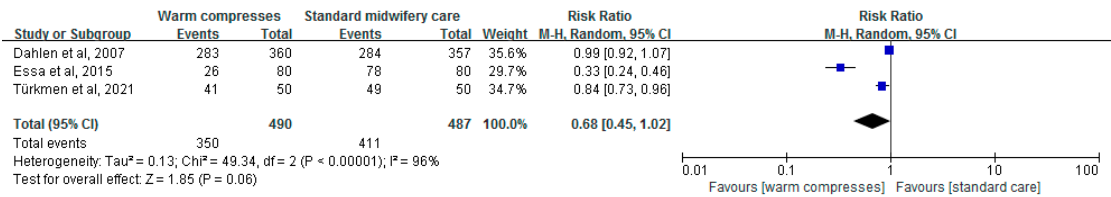

Supplement: Supplementary file 1 [file healthcare-12-00702-s001.zip › Figure S2. Effect of warm compresses on perineal lacerations requiring suture.pdf]
